# Inequalities in the utilisation of maternal health Care in Rural India: Evidences from National Family Health Survey III & IV

**DOI:** 10.1186/s12889-020-08480-4

**Published:** 2020-03-20

**Authors:** Balhasan Ali, Shekhar Chauhan

**Affiliations:** International Institute for Population Sciences, Govandi station Road, Deonar, Mumbai 400088 India

**Keywords:** Inequalities, India, Rural, Decomposition, Concentration index

## Abstract

**Background:**

Since the implementation of National Rural Health Mission (NRHM) in 2005, Maternal Mortality Ratio has significantly declined in India through a noticeable improvement in maternal health care services. However, India did not succeed to achieve the target of millennium development goal to reduced maternal mortality ratio by 2015. Also, there is substantial inequality exist at the regional, geographic, economic, and social level, and various socioeconomic factors contribute to the significantly large share in inequality in utilisation of maternal health care in India.

**Methods:**

Using data from the National Family Health Survey (2005 and 2015), this study examined the degree of inequality exist in maternal health care namely full antenatal care (full ANC), skilled attendants at birth (SBA), and postnatal care (PNC) in rural India. Descriptive statistics, concentration index (CI), and Wagstaff decomposition method have been performed to understand the pattern of maternal health care utilisation, and to explain the extent of inequality in maternal health care utilisation.

**Results:**

The study revealed that a substantial gap across socioeconomic groups exist in utilisation of maternal health care has significantly reduced in rural India during 2005–16. The results found a noticeable improvement in maternal health care utilisation, especially in utilisation of skilled attendants at birth (SBA). During this decade, the concentration index for SBA showed a significant decline from 0.28 in 2005–06 to 0.09 in 2015–16, while that of full ANC declined from 0.47 to 0.32 over the same period, and reduction of inequality in full ANC was least. Further, the results of decomposition analysis suggested that secondary and higher education, mass media exposure, and scheduled tribe contributed a significant share to the inequality.

**Conclusion:**

The exposure to mass media is the most significant contributor to inequality, and hence, there is a need for broad dissemination of awareness regarding maternal health care schemes in rural parts of country. Based on findings of study, it is suggested that health scheme related to maternal and child health care under NRHM be continued and focused for lower socioeconomic groups and marginalized mothers to reduce maternal health services inequality, particularly in the component of full ANC.

## Background

The gap in the risk of maternal deaths between developed and developing countries is considered as the most significant health divide in the world [1]. Maternal healthcare remains a major challenge to the global public health system, especially in developing countries [2]. Among the developing countries, India contributes to about 27 million births per year in the world and accounts for 20% of global maternal deaths [3]. Although maternal mortality has declined substantially in the last one decade, from 212 in 2007–09 [4] to 178 in 2010–12 [5] and further to 130 in 2014–16 [6], significant socio-economic differences still persist. A study [7] using the data for three time-periods from 1992 to 2006 confirmed that the utilization of maternal health care varied significantly with the economic status of the mothers in India. Not only the economic status of mother, but also the community and district-level factors are associated with the use of maternal health-care services in India [8]. In India, nearly 26% [9] of the population constitute women of reproductive age (15–49 years). These women are exposed to the risk of pregnancy and childbearing, and under existing socioeconomic differences [10] and the inequality in medical health facilities, these women are at a higher risk of morbidity and mortality resulting from the pregnancy-related issues. An improvement in the levels of Antenatal care, skilled attendants at birth, and Post-natal care among women are desirable to lower down the maternal mortality.

However, the elements of antenatal care in the Indian health system are since long; the progress is dismal. It was during the third five-year plan (1961–66) that the recruitment of Auxiliary Nurse Midwives (ANMs) provided the rural women access to some elements of antenatal care. Maternal and child health services became an integral part of the Indian health system during the fifth 5 year plan (1976–79), when maternal and child health services were integrated with the family planning services. In the rural areas of India, ANMs are responsible for providing antenatal care, but the access and utilization of these services is a matter of debate. According to the 2005–06 National Family Health Survey (NFHS- 3), only 12% of the mothers reported receiving full antenatal care for the last birth in the 5 years before the survey; the same has increased to nearly 21% as per NFHS-4 (2015–16). The increase is very minimal, and the scenario of full antenatal care is still very gloomy in India. According to the 2005–06 National Family Health Survey (NFHS- 3), about 44% of the mothers reported receiving antenatal care during the first trimester for the last birth in the 5 years before the survey; the same has increased to nearly 59% as per NFHS-4 (2015–16). This lack of improvement occurred despite governmental and non-governmental efforts to strengthen service delivery. Various studies in India have attributed the socio-economic differentials in antenatal care utilization to a combination of poor access to health services, low education levels, and poor demand [11–13]. A study [7] found that utilization of SBA significantly varied by the place of residence, and state of residence in India. The study further found the economic inequalities in the utilization of skilled birth attendance, but it concluded that economic inequalities are more prevalent in the use of prenatal care than skilled birth attendance in India.

Promoting antenatal care and skilled attendance at birth is not enough to improve maternal and child health. Postnatal care is as important as the other two factors discussed above. The importance of postnatal care can be understood by the fact that more than two-thirds of new-born deaths occur by the end of the first week after birth, with up to one-half of all deaths occurring in the first 24 h of birth [14]. Even in India, about 39% of neonatal deaths occur on the first day of life [15]. The WHO (1998) guidelines [16] recommended postnatal visits within 6 to 12 h after birth, and follow-up visits from 3 to 6 days, at 6 weeks, and then at 6 months. In a general trend, postnatal care has been limited in South Asia [17–19] and particularly in India. According to the 2005–06 National Family Health Survey (NFHS- 3), only 35% of the mothers reported receiving postnatal check-up within 2 days of their recent birth; the same has increased to nearly 62% as per NFHS-4 (2015–16). Postnatal care has not been studied to the desired extent in India, and only a few studies are available in India related to postnatal care; a study conducted in Madhya Pradesh [8], a rural-based study [20], and a study conducted in South India [11]. However, these studies did not explicitly addressed the socio-economic inequalities in postnatal care; these studies paved the way for further studies. A study [21] investigated the association between postnatal care and neonatal mortality in India.

The objective of this study is to quantify the prevailing inequalities in the utilization of maternal health care in rural India. The study looks at the factors contributing to the inequalities in the utilisation of maternal health care.

## Methods

This study is based on cross-sectional survey data available from the third (NFHS-3, 2005–06) and fourth (NFHS-4, 2015–16) round of National Family Health Survey. NFHS is a large-scale household survey that provides information on various indicators including maternal and child health in Inia. NFHS is a country representative survey of ever-married women, and the sample size is representative at the state level. The NFHS follows a multistage stratified random sampling design. In NFHS, primary sampling units are drawn by applying probability proportional to size and systematic sampling methods.

### Sample size

The total sample size of NFHS-4 is 699,686 women aged 15–49 years and sample size of NFHS-3 is 124,385 women. To carry out this study, women in rural areas are taken. NFHS-4 has 494,951 women in rural area and NFHS-3 has 67,424 women in rural areas. Out of this rural sampled women, only women with last birth have been taken for analysis purpose. Our outcome variables are Full Antenatal care, Skill attendant at birth, and postnatal care. For each of the three outcome variable, sample size is different. The table below depicts the sample size for NFHS-3 and NFHS-4.

**Table:** 

Sample Size			
	Full Antenatal Care	Skill Attendant at Birth	Postnatal care
NFHS-3	21,360	32,072	22,323
NFHS-4	138,945	198,116	142,983

The sample size may vary from NFHS reports because of different sample weight. We have used average weight (aw) while NFHS reports used individual weights. It is to be noted that by using a different weight, the results do not differ.

### Outcome variables

This study uses three indicators of maternal health to study inequality. The three indicators used in this study are full antenatal care (ANC), skilled birth attendants (SBA), and postnatal care (PNC). All the three outcome variables are based on the deliveries occurred to a woman in 5 years preceding the NFHS survey in both the rounds.

The definition used in calculating each of the variables is as follows:

#### Full antenatal care

Those women who had four or more visits for ANC, had at least two tetanus injection and consumed 100 IFA tablets/ syrup for the most recent birth is considered as having full antenatal care.

#### Skill attendants at birth

Those women who had their deliveries conducted either in a medical institution or at home assisted by a skilled person (doctor/nurse/Lady Health Visitor (LHV)/ Auxiliary Nurse Midwives (ANM). Only the most recent birth that occurred to women during the last 5 years preceding the survey is considered.

#### Postnatal care

Those women who went for a check-up to any health facilities within 48 h of delivery for their most recent birth.

#### Explanatory variable

The explanatory variables used are, age in years (15-24, and, 25-34, 35-49), Birth order (1, 2, 3 and above), educational status of the mother (no education, primary education, secondary education, and higher education), mass media exposure (No, Yes), caste (Scheduled Caste (SC), Scheduled Tribe (ST), Other Backward Caste (OBC), and Others), Religion (Hindu, Muslim, others), and wealth index.

For measuring state-wise inequality in Full ANC, SBA, and PNC, the states have been given the following codes: Kerala (KE), TamilNadu (TN), Andhra Pradesh (AP), Karnataka (KA), Jammu & Kashmir (JK), Punjab (PJ), Gujarat (GJ), Sikkim (SK), Maharashtra (MH), West Bengal (WB), Himachal Pradesh (HP), Rajasthan (RJ), Haryana (HR), Odisha (OD), Tripura (TR), Mizoram (MZ), Manipur (MN), Madhya Pradesh (MP), Bihar (BH), Chhattisgarh (CH), Jharkhand (JH), Uttar Pradesh (UP), Uttarakhand (UK), Assam (AS), Nagaland (NA), Arunachal Pradesh (AR), Meghalaya (MG).

### Statistical approach

For this study, we have used the wealth index as a proxy measure of household economic status as NFHS does not have direct information on income. NFHS provides five categories of wealth index namely; poorest, poorer, middle, richer, and richest. We have used Principal Component Analysis (PCA) to produce the factor score for the continuous variable for each household. For the calculation of Concentration Index (CI), we have divided the wealth index into five equal quintiles, and for decomposition analysis, we have used the variable of continuous wealth index. For the decomposition analysis, we have generated a dummy category to be used as a reference category for each variable; age of women (15–24 years), Birth order (first), Women’s education (no education), Caste (Others), Religion (Others), and mass media exposure (No).

#### Concentration index (CI)

The concentration curve is defined as twice the area between the concentration curve and the line of equality. In the present context of the study, the concentration index has been used to measure the health-related income inequalities in full antenatal care, skilled attendants at birth, and postnatal care. The CI curve depicts the cumulative percentage of the health outcome variable (y-axis) for the cumulative percentage of the sample population by the socio-economic status on the x-axis. These curves can be used to identify whether socio-economic inequality in outcome variable exists or not and whether it is more pronounced at one point of time than another or in one country than in another.

When the curve lies above the line of equality meaning that index has a negative value, it depicts the disproportionate concentration of the outcome variable among the poor, and when CI curve lies below the line of equality meaning that index has a positive value, it demonstrates the disproportionate concentration of the outcome variable among the better-off. The value of the concentration index varies from − 1 to + 1, where a value of 0 (Zero) depicts no socio-economic inequality. A positive value of CI depicts the pro-rich inequality and vice-versa. The value of CI quantifies the extent of socio-economic inequality. The larger the absolute value, the greater the inequalities.

CI can be depicted as follows:

1 $$ \boldsymbol{C}=\frac{\mathbf{2}}{\boldsymbol{\mu}}\boldsymbol{\operatorname{cov}}\left({\boldsymbol{y}}_{\boldsymbol{i}},{\boldsymbol{R}}_{\boldsymbol{i}}\right) $$

Where, C is the concentration index; ***y***_***i***_ is outcome variable index; ***R*** is the fractional rank of individual ***i*** in the distribution of socio-economic position; ***μ*** is the mean of the outcome variable of the sample and ***cov*** denotes the covariance.

For decomposing the concentration index, we have used Wagstaff decomposition analysis. Wagstaff’s decompostion demonstrated that the concentration index could be decomposed into the contributions of each factor to the income-related inequalities. Each contribution is the outcome of the sensitivity of health concerning that socio-economic factor and the extent of income-related inequality in that factor. Based on the linear regression relationship between the outcome variable *y*_*i*_, the intercept *α*, the relative contribution of *x*_*ki*_ and the residual error ***ε***_***i***_ in the eq. 2,

2 $$ {y}_i=\alpha +\sum {\beta}_k{x}_{ki}+{\varepsilon}_i $$

Where *ε*_*i*_ is an error term, given the relationship between *y*_*i*_ and *x*_*ki*_ in the eq. 2, the CI for y (C) can be rewritten as in eq. 3:

3 $$ C=\sum \left(\frac{\beta_k{\overline{x}}_k}{\mu}\right){C}_k+\frac{GC\varepsilon}{\mu }/\mu $$

where *μ* is the mean of *y*_*i*_, ${\overline{x}}_k$ is the mean of *x*_*k*_, *β*_*k*_ is the coefficient from a linear regression of outcome variables, *C*_*k*_ is the concentration index for *x*_*k*_ (defined analogously to *C*, and *GC*_ε_ is the generalized concentration index for the error term (*ε*_*i*_).

Equation (3) shows that C is the outcome of two components: First, the determinants or ‘explained’ factors, which are equivalent to the weighted accumulation of the concentration indices of the regressor, where one unit change in the outcome variable is to be associated with the one unit change in the explanatory variable. The explained factors indicate that the proportion of inequalities in the outcome (full ANC, SBA, PNC) variable is explained by the selected explanatory factors, i.e., *x*_*k*_.

Second, a residual or ‘unexplained’ factor $\Big(\frac{\boldsymbol{GC}\boldsymbol{\upvarepsilon }}{\boldsymbol{\mu}}/\boldsymbol{\mu}$**),** indicating the inequality in health variable that cannot be explained by selected explanatory factors across various socioeconomic groups.

## Results

Table 1 shows the percentage distribution of utilization of full antenatal care, skilled attendants at births and postnatal care for the two time-periods from 2005 to 06 to 2015–16 by socio-economic characteristics of the rural women. However, the utilization of all the three determinants of maternal health-care namely; full antenatal care, skill attendants at birth, and postnatal care has increased among the women from all the socio-economic groups over the two periods from 2005 to 06 to 2015–16, significant differences still persist by background characteristics. With the increase in age and the birth order, the utilization of all the three services goes on decreasing. The utilization of full ANC, SBA, and PNC has almost doubled among the women of the age group 15–24 between the two time-periods. The utilization of SBA goes on decreasing with the increase in birth order, which suggests that women give utmost priority to their first child. However, the utilization of SBA among the women with 3+ births has increased threefold from 2005 to 06 to 2015–16; the utilization of SBA remains high for the first birth order in both the time periods. A higher percentage of women who were richest, highly educated, and had access to mass media utilized the maternal health care services than their counterparts.

**Table 1:** percentage distribution of the utilisation of full antenatal care, skilled attendants at birth and post-natal care by selected socio-economic characteristics among rural women in India, 2005–16

Background Variables	NFHS-3			NFHS-4		
	Full ANC	SBA	PNC	Full ANC	SBA	PNC
Age						
15–24	8.1	42.7	28.2	17.0	82.8	61.3
25–34	9.1	35.4	25.5	16.2	77.2	57.9
35–49	3.8	23.1	15.2	10.2	63.4	46.0
Birth Order						
1	13.6	56.3	56.3	21.3	87.0	67.0
2	10.7	43.1	43.1	18.9	80.3	62.1
3+	4.1	22.9	22.9	8.5	65.8	46.3
Mother’s Educational Status						
No education	2.6	23.1	13.3	6.8	64.8	43.7
Primary education	7.9	40.5	26.9	12.8	75.4	54.6
Secondary education	16.6	63.3	45.5	21.2	87.6	66.9
Higher education	39.4	90.4	73.4	32.3	94.3	76.4
Media Exposure						
No	2.7	22.5	12.6	5.6	64.6	41.9
Yes	11.4	47.1	33.5	20.7	84.9	65.5
Caste						
Schedule caste	5.5	33.6	22.9	15.4	78.4	58.2
Schedule tribe	4.7	22.0	14.9	14.4	69.5	51.9
OBC	8.3	38.8	25.6	15.3	79.2	58.3
Others	11.7	47.3	33.9	19.3	81.6	62.8
Religion						
Hindu	8.2	38.8	26.4	16.2	79.9	79.9
Muslim	6.9	27.1	18.5	11.4	66.6	66.6
Others	9.8	49.2	36.8	24.1	79.4	79.4
Wealth Index						
poorest	2.3	18.8	10.6	6.2	64.1	42.2
poorer	4.4	30.8	18.6	13.3	78.2	56.0
middle	9.0	46.6	30.9	21.1	87.4	66.9
richer	16.6	62.1	45.1	27.4	91.4	73.8
richest	31.8	83.8	68.5	32.3	94.6	79.8

**Fig. 1:**
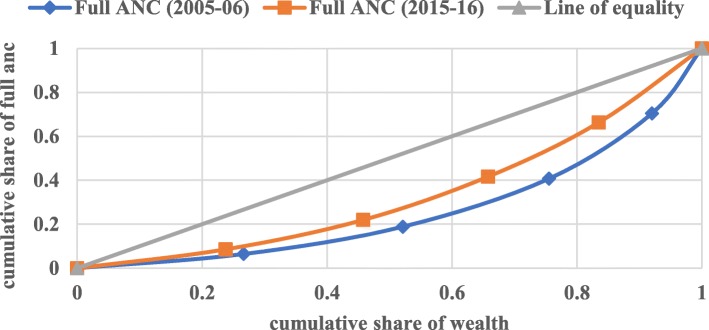
Concentration Curve for Full ANC in India, 2005–06 & 2015–16. Legend: Full ANC (2005–06), Full ANC (2015–16), Line of equality

**Fig. 2:**
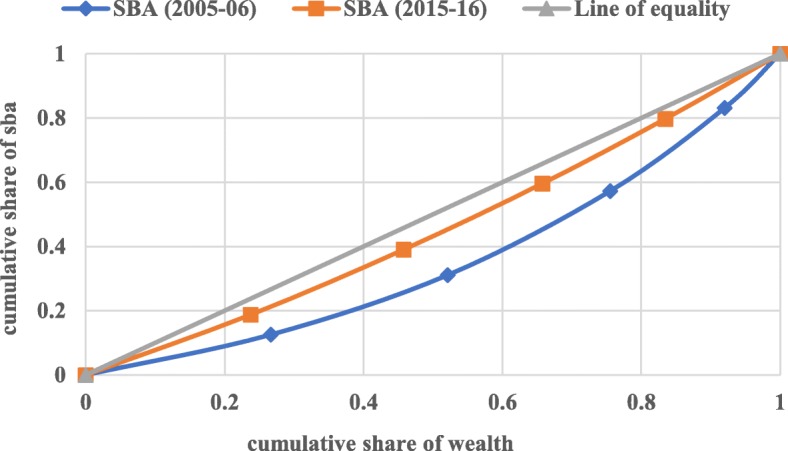
Concentration Curve for SBA in India, 2005–06 & 2015–16. Legend: SBA (2005–06), SBA (2015–16), Line of equality

**Fig. 3:**
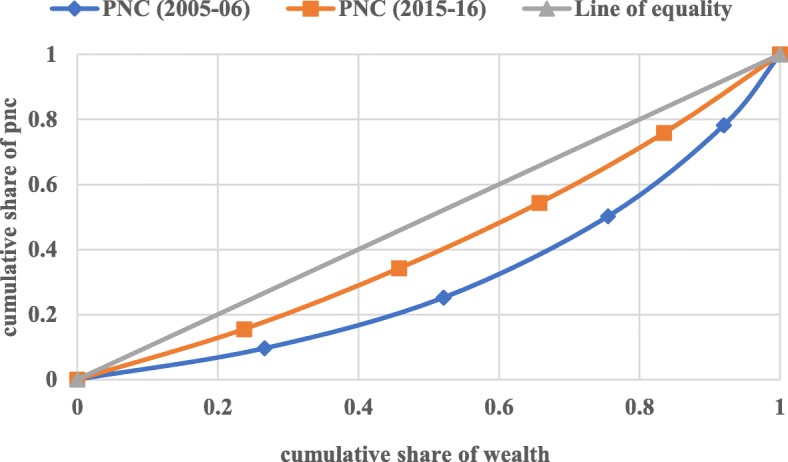
Concentration Curve for PNC in India, 2005–06 & 2015–16. Legend: PNC (2005–06), PNC (2015–16), Line of equality

Figures 1, 2, and 3 depict the concentration curve for inequality in the utilization of full ANC, SBA, and PNC respectively for India for the year 2005–06 and 2015–16. The result of the concentration curve suggests a reduction in inequality for all the three maternal health-care services. In a decade from 2005 to 06 to 2015–16, However, there is a substantial reduction in the inequality in full antenatal care, skilled attendants at birth, and postnatal care, the inequality in the utilization of SBA had reduced much faster than the full ANC and PNC. The inequality in the utilization of full ANC is still a cause for concern in India.

**Fig. 4:**
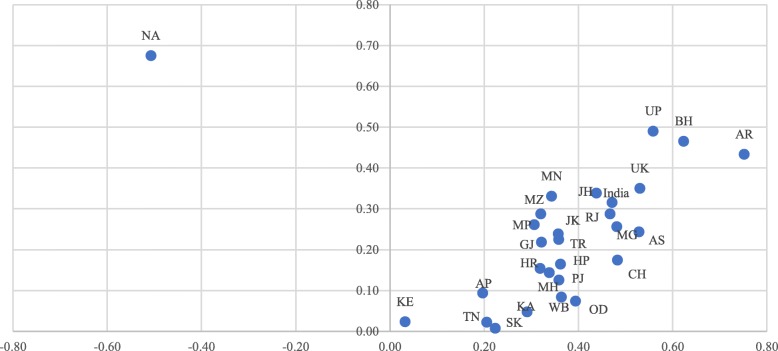
Scatter plot of CI for full ANC in India and its states between 2005 and 06 and 2015–16. Legend: States

Figure 4 shows the result of variation in the concentration index for the utilization of full ANC in the states of India from 2006 to 06 to 2015–16. The CI for full ANC has been reduced by 15 points in India from 0.47 in 2005–06 to 0.32 in 2015–16. The inequality for full ANC has reduced by about 30 points between the two time-periods in the states of Arunachal Pradesh, Chhattisgarh, and Odisha. The inequality in the utilization of full ANC has reduced from 2005 to 06 to 2015–16 to a substantial level in all the states of India except Nagaland, Uttar Pradesh, Bihar, and Arunachal Pradesh. The inequality in the utilization of full ANC is very low in the states of Sikkim, Kerala, TamilNadu, and Karnataka. The CI for most of the state varies between 0.10–0.30 in 2015–16 whereas, in 2005–06, the CI for most of the states varied between 0.30–0.60. Although the states of Nagaland, Uttar Pradesh, Bihar, Arunachal Pradesh, Uttarakhand, Jharkhand, Manipur, and Rajasthan have shown some improvements in CI during 2005–16, the level of inequalities remained large.

**Fig. 5:**
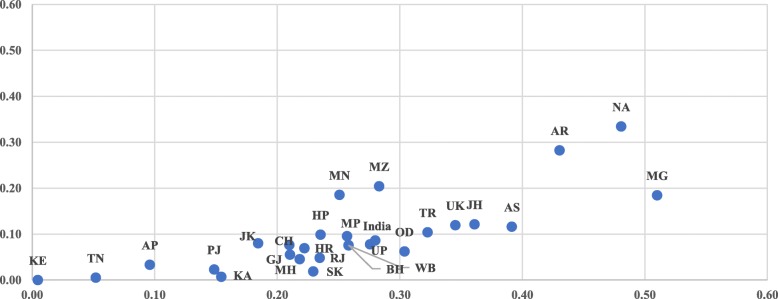
Scatter plot of CI for SBA in India and its states between 2005 and 06 and 2015–16. Legend: States

Figure 5 shows the result of variation in the concentration index for the utilization of SBA in the states of India from 2006 to 06 to 2015–16. The inequality in the utilization of SBA has reduced by 19 points, from 0.28 in 2005–06 to 0.09 in 2015–16, in India. In the states of Meghalaya, Assam, Odisha, Jharkhand, Uttarakhand, Tripura, Sikkim, and Uttar Pradesh, the inequality in the utilization of SBA has reduced by more than 20 points between the two time-periods. However the result shows that inequality in the utilization of skilled attendants at birth has reduced in all of the states in India; significant differences still persist at the state level. The inequality is as high as 0.33 in the state of Nagaland and as low as 0.01 in the state of TamilNadu and Karnataka during 2015–16. In the state of Kerala, there is no inequality in the utilization of skill attendants at birth during both the time-periods. The inequality in the utilization of SBA is least in the state of TamilNadu and Karnataka, and it is highest in the North-eastern states during 2015–16.

**Fig. 6:**
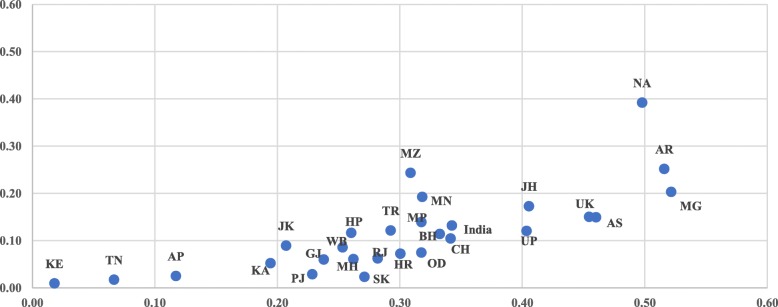
Scatter plot of CI for PNC in India and its states between 2005 and 06 and 2015–16. Legend: States

Figure 6 shows the result of variation in the concentration index for the utilization of PNC in the states of India from 2006 to 06 to 2015–16. The inequality in the utilization of PNC has reduced by 21 points, from 0.34 in 2005–06 to 0.13 in 2015–16, in India. In the states of Meghalaya, Assam, Uttarakhand, Uttar Pradesh, Arunachal Pradesh, Sikkim, Odisha, Chhattisgarh, Jharkhand, and Haryana, the inequality in the utilization of PNC has reduced by more than 22 points between the two time-periods. However, the result shows that inequality in the utilization of PNC has reduced in all of the states in India; significant differences still persist at the state level. The inequality is as high as 0.39 in the state of Nagaland and as low as 0.01 in the state of Kerala during 2015–16. For the time period 2015–16, the inequality in the utilization of PNC is highest in the state of Nagaland followed by Arunachal Pradesh, Mizoram, Meghalaya, Manipur, Jharkhand, Uttarakhand, and Assam, and least in the state of Kerala followed by TamilNadu, Sikkim, Punjab, Karnataka, Gujarat, Maharashtra, and Rajasthan.

Table 2 presents the estimates of marginal effect and decomposition analysis of inequality in the utilization of full antenatal care in India for the two time-periods. The decomposition analysis presents the contribution to the inequality in the utilization of full ANC as explained by the predictor variables namely; age in years, birth order, women’s education, caste, religion, and exposure to mass media. The marginal effect takes either a negative or a positive value; positive marginal effect indicates that the explanatory variable has a positive association with the maternal health-care outcomes and has a higher likelihood of utilization of the healthcare services and vice-versa. The value of the absolute contribution indicates the extent of inequality contributed by the explanatory variables. For instance, women who are educated and have exposure to mass media have more propensity to utilise full ANC during both the time-periods. A positive value of CI indicates that the women with the characteristic in question are highly represented among the rich and vice-versa. The positive value of an absolute contribution to CI indicates the pro-rich which means rich individuals use more health care services as compared to poor, and vice-versa. During 2005–06, the secondary and higher education of the mother is explaining about 69% of inequality in the utilization of full antenatal care. 20% of inequality is contributed by the 3+ birth order, and exposure to mass media is contributing about 12% of inequalities in the utilization of full ANC during 2005–06. During 2015–16, exposure to mass media is the main cause of inequality as it contributes about 40% to the inequalities. About 44% of the inequality in the utilization of full ANC is determined by the secondary and higher education of mother during 2015–16. During both the time-periods, 3+ birth orders, secondary and higher education level of the mother, and exposure to mass media is contributing highly to inequalities in the utilization of full ANC.

**Table 2:** Estimates of Marginal effect and decomposition analysis for contribution of explanatory factors to the inequality in full antenatal care among rural women in India, 2005–16

Explanatory variables	2005–06				2015–16			
	Marginal Effect	CI	Absolute Contribution to CI	Percentage Contribution to CI	Marginal Effect	CI	Absolute Contribution to CI	Percentage Contribution to CI
Age in years (25-34)	0.045	0.005	0.000	0.40	0.038	0.008	0.001	0.53
Age in years (35-49)	0.035	− 0.187	− 0.002	−2.39	0.048	− 0.244	− 0.004	−3.09
Birth order (2)	− 0.029	0.109	− 0.003	−3.19	− 0.022	0.074	− 0.002	−1.51
Birth order (3+)	− 0.070	− 0.151	0.020	19.62	− 0.087	− 0.220	0.025	18.75
Primary education	0.042	0.064	0.002	1.59	0.029	−0.103	− 0.002	− 1.36
Secondary education	0.114	0.414	0.049	48.71	0.081	0.242	0.034	25.30
Higher education	0.314	0.783	0.021	20.52	0.162	0.624	0.026	19.20
Schedule tribe	0.006	−0.356	− 0.001	−1.00	− 0.007	− 0.095	0.001	0.53
OBC	0.016	0.048	0.001	1.29	−0.003	− 0.299	0.001	0.38
Others	0.014	0.230	0.003	3.09	−0.010	0.044	−0.001	− 0.60
Hindu	0.022	−0.005	0.000	− 0.30	− 0.053	− 0.006	0.001	0.75
Muslim	0.034	−0.025	−0.001	− 0.50	− 0.066	− 0.037	0.001	0.98
Media exposure	0.026	0.191	0.012	12.15	0.090	0.223	0.053	40.14
**Explained CI**			**0.100**	**100**			**0.133**	100
**Total CI**			**0.471**				**0.316**	
**Residual**			**0.371**				**0.183**	

Table 3 presents the estimates of marginal effect and decomposition analysis of inequality in the utilization of skill attendants at birth in India for the two time-periods. Like full ANC, the explanatory variables defining the inequalities in the utilization of SBA are almost the same. The increasing age of the women, increasing birth order, lower education levels, and non-exposure to the mass media are positively associated with the inequalities in the utilization of SBA. The result found that as the birth order increases the likelihood of utilization of SBA decreases among women. The increase in education levels is accompanied by an increased likelihood of utilizing SBA in both the time-periods. The religion-wise and caste-wise inequalities in the utilization of SBA are low, but persistent. The inequalities as explained by the exposure to the mass media in the utilization of SBA has increased from 14 percentage during 2005–06 to 34 percentage during 2015–16. The inequalities due to secondary and higher levels of education of mothers have declined from 53% in 2005–06 to 42% during 2015–16. During both the time-periods, about 20% of the inequality is determined by the 3+ birth order.

**Table 3:** Estimates of Marginal effect and decomposition analysis for contribution of explanatory factors to the inequality in skilled attendants at birth among rural women in India, 2005–16

Explanatory variables	2005–06				2015–16			
	Marginal Effect	CI	Absolute Contribution to CI	Percentage Contribution to CI	Marginal Effect	CI	Absolute Contribution to CI	Percentage Contribution to CI
Age in years (25-34)	0.047	0.005	0.001	0.18	0.011	0.008	0.000	0.11
Age in years (35-49)	0.047	−0.187	−0.003	−1.14	− 0.023	−0.244	0.002	1.09
Birth order (2)	− 0.116	0.109	− 0.013	−4.58	− 0.051	0.074	− 0.005	−2.51
Birth order (3+)	− 0.237	− 0.151	0.066	23.50	− 0.123	− 0.220	0.035	19.17
Primary education	0.112	0.064	0.004	1.49	0.056	−0.103	− 0.004	−1.91
Secondary education	0.276	0.414	0.119	42.10	0.126	0.242	0.052	28.49
Higher education	0.479	0.783	0.032	11.18	0.157	0.624	0.025	13.45
Schedule tribe	−0.088	−0.356	0.015	5.36	−0.013	− 0.095	0.001	0.65
OBC	0.044	0.048	0.004	1.28	−0.080	− 0.299	0.013	6.86
Others	0.069	0.230	0.015	5.40	0.000	0.044	0.000	0.00
Hindu	−0.070	−0.005	0.001	0.39	0.000	−0.006	0.000	−0.22
Muslim	−0.150	−0.025	0.002	0.82	−0.075	−0.037	0.002	0.82
Media exposure	0.085	0.191	0.040	14.02	0.105	0.223	0.062	33.99
**Explained CI**			**0.282**	**100**			**0.184**	**100**
**Total CI**			**0.280**				**0.086**	
**Residual**			**−0.002**				**−0.097**	

Table 4 presents the estimates of marginal effect and decomposition analysis of inequality in the utilization of postnatal care in India for the two time-periods. Increasing age of the mother and increasing birth order have a negative association with the utilization of PNC services. Increasing education levels and exposure to mass media are positively associated with the utilization of PNC services. In both the time-periods, exposure to mass media is the biggest contributor to the inequality in the utilization of PNC among women. During 2005–06, Exposure to mass media, along with the secondary and higher level of education contribute about 70% inequality in the utilization of PNC among the women, the same has been increased to 80% during 2015–16. However, the inequalities in the utilization of PNC have declined from .34 in 2005–06 to .13 in 2015–16 as explained by the total CI; it remains unequally distributed among the various predictors.

**Table 4:** Estimates of Marginal effect and decomposition analysis for contribution of selected socio-demographic factors to the inequality postnatal care among rural women in India, 2005–16

	2005–06				2015–16			
Explanatory variables	Marginal Effect	CI	Absolute Contribution to CI	Percentage Contribution to CI	Marginal Effect	CI	Absolute Contribution to CI	Percentage Contribution to CI
Age in years (25-34)	0.066	0.005	0.001	0.31	0.035	0.008	0.001	0.30
Age in years (35-49)	0.062	−0.187	−0.004	−1.84	0.022	−0.244	− 0.002	−0.90
Birth order (2)	−0.059	0.109	−0.007	−2.89	− 0.044	0.074	− 0.004	− 1.99
Birth order (3+)	− 0.186	− 0.151	0.052	22.80	− 0.128	−0.220	0.037	18.25
Primary education	0.094	0.064	0.004	1.53	0.053	−0.103	−0.003	−1.64
Secondary education	0.225	0.414	0.097	42.45	0.121	0.242	0.050	25.06
Higher education	0.446	0.783	0.029	12.82	0.170	0.624	0.027	13.28
Schedule tribe	−0.056	−0.356	0.010	4.20	−0.015	− 0.095	0.001	0.65
OBC	0.015	0.048	0.001	0.53	−0.056	−0.299	0.009	4.48
Others	0.034	0.230	0.007	3.24	−0.011	0.044	−0.001	− 0.45
Hindu	−0.056	− 0.005	0.001	0.39	−0.034	− 0.006	0.001	0.35
Muslim	−0.088	−0.025	0.001	0.61	−0.111	−0.037	0.002	1.14
Media exposure	0.078	0.191	0.036	15.84	0.141	0.223	0.083	41.47
**Explained CI**			**0.229**	**100**			**0.201**	**100**
**Total CI**			**0.343**				**0.132**	
**Residual**			**0.114**				**−0.069**	

## Discussion

This study deepens our awareness about the wide-ranging and persistent factors of inequality in the utilization of maternal healthcare services. We attempted to measure the inequalities in the utilization of maternal health care services among rural women in India by capturing the dimension of full antenatal care, skill attendants at birth, and postnatal care. We found clear evidence of inequalities in the utilization of full ANC, SBA, and PNC at the state level as well as by the various explanatory variables. India is a country with huge diversity, and the states differ enormously in terms of geography, demography, language, and social norms. Regional disparities among India’s major states are both large and persistent [22]. A study found the strong indications of a pervasive increase in economic inequality during the nineties in India [23]. A study [24] examined the pattern of regional inequalities in India during 1970–92 and found the inter-state inequalities to be rising in India in the economic sphere. Another study [25] analyzed inequality and poverty in India within the context of caste-based discrimination and found the caste-driven inequalities in India. A study [26] explored the patterns of economic discrimination faced by Dalits and minorities like Muslims in India and analyzed the discrimination in access to education and primary health-care services.

### Inequalities in the utilization of full ANC, SBA & PNC

We have found clear evidence of inequalities in the utilization of full antenatal care. Mother’s education is one of the important factors contributing to the inequality in the utilization of full ANC. The utilization of full antenatal care increases with the increase in the levels of mother’s education. Higher educated women are more likely to use full ANC than uneducated women. A study [10] on rural adolescent women in India found that women with higher education were three times more likely to utilize the full antenatal care than uneducated women. Many studies conducted in India [27–29], and other countries [30–32] have found that mother’s education is one of the most important determinants of antenatal care utilization. High education standards among women enhance the likelihood of communication with the husband and other family members on health-related issues [12], and this helps in the higher utilization of antenatal care services. Educational attainment is critical in imparting the feelings of self-worth and self-confidence which are critical in bringing the changes in health-related behaviour [33]. Accumulation of wealth in the household is another factor contributing to the inequality in the utilization of full antenatal care. Our result suggests that the women in richest wealth quintile are more likely to utilize the full antenatal care than the women in poorest wealth quintile. The utilization of full antenatal care is higher among the subsequent wealth quintile as compared to the previous category of wealth quintile. This evidence is consistent with the other studies [34–36]. Another study in the Indian context also found that women in the richest wealth quintile were more likely to use full antenatal care than the poorest women [10].

We have found that social factors like religion and caste also play an important role in promoting inequalities in the utilization of antenatal care services. The study found that Muslim women and Scheduled caste women are less likely to utilize the antenatal care services. Our findings are in line with other studies [10, 12]. A study [13] conducted in rural North India found the widespread caste-wise inequalities in the utilization of antenatal care; another study [37] found the same level of caste-wise inequalities in South India. Our study highlights the role of birth order in determining the inequality in the utilization of antenatal care. The higher the birth order, the lower will be the antenatal care. A study [38] highlighted the limited care during the antenatal period for the second and higher order births than for the first birth. Another study [12] have also shown this relationship between birth order and the utilization of maternal healthcare services. A study found higher parity to be associated with the reduced use of antenatal care [13]. This can be attributed to the fear of first birth. Women, delivering the baby for the first time, are more cautious about their pregnancy and are more likely to face difficulties during the labour than women with high parity [39]. A study concludes that women with higher parity develop confidence after delivering the first birth, and they use their experience and knowledge from previous pregnancies [10], thus limiting the use of maternal healthcare services for the higher birth order.

We have found that exposure to mass media has a positive association with the utilization of antenatal care services. Our study accords with other studies from Bangladesh [40], Nepal [41], and India [42, 43] where exposure to mass media had positive influence with an increased antenatal care visit. A study [12] found that women with a high degree of exposure to mass media were more likely to received antenatal check-up. We found the inter-state inequalities in the utilization of full antenatal care. The utilization of full antenatal care is higher among the women in the Southern states of India as compared to women in other states of India. A study also found the regional variation where full antenatal care utilization was found to be less likely in other states of India as compared to the Southern states of India [10]. Various studies in India found that the utilization of antenatal care services is higher among the mothers in the Southern states of India as compared to women in other states [27].

Our study found the clear evidence of inequalities in the utilization of skilled attendants at birth. Increasing age of the mother and increasing birth order have a negative association with the utilization of SBA, whereas, increasing education level among women and exposure to mass media have a positive association with the utilization of SBA. The inequalities in the use of SBA are more prominent among uneducated women as compared to women with higher education and among the women who have no access to mass media than to the women who have access to mass media. About one-third of the inequality in the utilization of SBA is contributed by the exposure to mass media among women. Thus it can be understood that mass media exposure is the main contributor to the inequality in the utilization of SBA. A study conducted in Nepal found that mass media information were partially successful in increasing the use of maternal health services [44]. Another study based on Uttarakhand, a state in India, found that a higher percentage of women with exposure to mass media opted for safe delivery than women who were not exposed to mass media [45]. The exposure to mass media is positively associated with the utilization of maternal healthcare services. The exposure to mass media mitigate against the cultural barriers for using healthcare services, and it is one of the important sources of information regarding the beneficial impact of care for maternal and child health [46].

The result of our study suggests that a higher percentage of richest women utilize SBA than the poorest women. Wealth is one of the strongest determinants of skilled birth attendant’s use, with the poor people being at a disadvantage [47]. Our result is consistent with other studies related to the inequalities in the utilization of SBA and wealth distribution [48]. Social factors like religion and caste are significant factors affecting utilization of skill attendants at birth. Our study found the religion-wise inequalities in the utilization of skill attendants at birth, where Muslim women are least likely to utilize this service. A study opined that the possibility of the purdah system may be a significant contributor to the low level of utilization of SBA among Muslim women [47]. Purdah system is a physical segregation of the sexes, where women are required to cover their bodies and conceal their form [49]. Our study also found caste-wise inequalities in the utilization of SBA, where lesser women from Scheduled Caste, Scheduled Tribes, and Other Backward Classes utilize the SBA. Previous studies have also shown that fewer women from SC, ST, and OBC had an attendant present at birth [12, 28, 50] as compared to women from other caste. Our study found that with the higher order of birth, the utilization of SBA declines which is consistent with other study [47].

The findings show women’s age, birth order, education level, and mass media exposure as significant factors of inequality affecting postnatal care utilization. The results found that with an increase in women’s age and birth order, the likelihood of utilization of postnatal care decreases; whereas with an increase in education level, wealth, and access to mass media exposure, the likelihood of utilization of postnatal care increases. The result found that mass media exposure is the biggest contributor to the inequalities in the utilization of postnatal care. A study conducted in Indonesia found that low household wealth index and low maternal education levels are the factors affecting poor response to postnatal care [51]. A study in India found that women’s education, social group, mass media exposure, wealth quintile, birth order are the significant factors affecting postnatal care utilization [52]. Our study found that religion and caste are also important factors in determining the inequalities in the utilization of postnatal care. A study found that women from Scheduled Caste, Scheduled Tribes, and Other Backward Classes were less likely to seek postnatal care [52].

## Conclusion

By our findings, we suggest that specific efforts should be made to provide basic maternal healthcare services to the women of lower socio-economic status. Both the education levels and wealth are positively associated with the utilization of ANC, SBA, and PNC, and hence economic and educational improvement of the poor mothers would have a positive effect on reducing the prevalent inequalities. The exposure to mass media is the largest contributor to the inequality, and hence there is a need for wide dissemination of mass media in rural parts of the country. Not only exposure to mass media, but the public health system in terms of infrastructure, human resources, and management needs to be upgraded as exposure to mass media will soon be followed by the widespread utilization of maternal healthcare services. Mother’s education is another factor that is contributing highly to the inequalities in the utilization of maternal healthcare services. There is a need to disseminate the importance of women’s education in rural parts of India as despite having access to the schooling parents do not deem it necessary to educate their daughters.

The state-wise differential in the use of maternal healthcare services is widely prevalent. The north-eastern states need to be relooked along with the states like Uttar Pradesh, Bihar, and Jharkhand. It is highly recommended that the government of these states shall put an extra effort to provide maternal healthcare to the women. However, various schemes and policies going on for the improvements in the levels of maternal healthcare utilization in these states, how effectively they are being implemented is a cause for concern. It was expected that the key strategies adopted under National Rural Health Mission (NRHM) would improve the overall quality of health services in India as a whole, it failed to bring equality in the states of India in terms of service delivery. The NRHM (2005) prioritized North Indian states to improve maternal healthcare [27], but it failed to achieve the desired improvements as in rural areas as the program hit badly by the absence of doctors/health providers, low levels of skills, shortage of medicines, and inadequate supervision. There is a need to monitor the ongoing programs more closely.

## Data Availability

The study utilises secondary source of data which is freely available in public domain through http://iipsindia.org. The necessary ethical approval has been taken by the respective organisations involved in the data collection process.

## Abbreviations

ANM: Auxiliary Nurse Midwives
NFHS: National Family Health Survey
ANC: Antenatal Care
SBA: Skilled Birth Attendants
PNC: Postnatal Care
WHO: World Health Organization
IFA: Iron-Folic Acid (Tablet)
LHV: Lady Health Visitor
SC: Scheduled Caste
ST: Scheduled Tribe
OBC: Other backward Class
PCA: Principal Component Analysis
CI: Concentration Index
NRHM: National Rural Health Mission
