# Correction to: Inequalities in the utilisation of maternal health Care in Rural India: Evidences from National Family Health Survey III & IV

**DOI:** 10.1186/s12889-021-10799-5

**Published:** 2021-05-31

**Authors:** Balhasan Ali, Shekhar Chauhan

**Affiliations:** International Institute for Population Sciences, Govandi station Road, Deonar, Mumbai, 400088 India

**Correction to: BMC Public Health 20, 369 (2020)**

**https://doi.org/10.1186/s12889-020-08480-4**

It was highlighted that in the original article [1] current reference [2] is incorrect and should be replaced by a research article by Aliyu, B.M. (2018) [2]. Furthermore, that a research article by Rani et al. (2007) was not cited [3] in the Background section of the article. Finally, an incorrect definition was included of concentraction index curves in the Concentration Index section of the article which should be updated to: ‘The concentration curve plots the cumulative percentage of the health variable (y-axis) against the cumulative percentage of the population, ranked by living standards, beginning with the poorest, and ending with the richest (x-axis)’ [4]. The authors would like to sincerely apologise for this oversight. The original article has been updated
